# PREDICT HF: Risk stratification in advanced heart failure using novel hemodynamic parameters

**DOI:** 10.1002/clc.24277

**Published:** 2024-06-05

**Authors:** Nicole Cyrille‐Superville, Sriram D. Rao, Jason P. Feliberti, Priyesh A. Patel, Kamala Swayampakala, Shashank S. Sinha, Eric I. Jeng, Rohan M. Goswami, David F. Snipelisky, Aubrie M. Carroll, Samer S. Najjar, Mark Belkin, Jonathan Grinstein, the FLIGHT Working Group

**Affiliations:** ^1^ Sanger Heart and Vascular Institute, Atrium Health Charlotte North Carolina USA; ^2^ Department of Medicine, Medstar Washington Hospital Center, Division of Cardiology Georgetown University Washington District of Columbia USA; ^3^ University of South Florida Heart and Vascular Institute, Transplant Cardiology Tampa Florida USA; ^4^ Inova Heart and Vascular Institute, Inova Fairfax Medical Campus Falls Church Virginia USA; ^5^ Department of Surgery, Division of Cardiovascular Surgery University of Florida Gainesville Florida USA; ^6^ Division of Transplant, Research and Innovation, Mayo Clinic in Florida Jacksonville Florida USA; ^7^ Section of Heart Failure & Cardiac Transplant Medicine, Cleveland Clinic Florida Weston Florida USA; ^8^ Department of Medicine Duke University School of Medicine Durham North Carolina USA; ^9^ Medstar Heart and Vascular Institute Baltimore Maryland USA; ^10^ Department of Medicine, Section of Cardiology University of Chicago Chicago Illinois USA

**Keywords:** heart failure, novel hemodynamics, prognostication

## Abstract

**Background:**

Invasive hemodynamics are fundamental in assessing patients with advanced heart failure (HF). Several novel hemodynamic parameters have been studied; however, the relative prognostic potential remains ill‐defined.

**Hypothesis:**

Advanced hemodynamic parameters provide additional prognostication beyond the standard hemodynamic assessment.

**Methods:**

Patients from the PRognostic Evaluation During Invasive CaTheterization for Heart Failure (PREDICT‐HF) registry who underwent right heart catheterization (RHC) were included in the analysis. The primary endpoint was survival to orthotopic heart transplant (OHT) or durable left ventricular assist device (LVAD), or death within 6 months of RHC.

**Results:**

Of 846 patients included, 176 (21%) met the primary endpoint. In a multivariate model that included traditional hemodynamic variables, pulmonary capillary wedge pressure (PCWP) (OR: 1.10, 1.04−1.15, *p* < .001), and cardiac index (CI) (OR: 0.86, 0.81−0.92, *p* < .001) were shown to be predictive of adverse outcomes. In a separate multivariate model that incorporated advanced hemodynamic parameters, cardiac power output (CPO) (OR: 0.76, 0.71−0.83, *p* < .001), aortic pulsatility index (API) (OR: 0.94, 0.91−0.96, *p* < .001), and pulmonary artery pulsatility index (OR: 1.02, 1.00−1.03, *p* .027) were all significantly associated with the primary outcome. Positively concordant API and CPO afforded the best freedom from the endpoint (94.7%), whilst negatively concordant API and CPO had the worst freedom from the endpoint (61.5%, *p* < .001). Those with discordant API and CPO had similar freedom from the endpoint.

**Conclusion:**

The advanced hemodynamic parameters API and CPO are independently associated with death or the need for OHT or LVAD within 6 months. Further prospective studies are needed to validate these parameters and elucidate their role in patients with advanced HF.

## Graphical abstract

(A) Improved risk stratification of advanced heart failure using the advanced hemodynamic parameters, aortic pulsatility index (API), and cardiac power output (CPO). The simultaneous incorporation of API and CPO into risk models defines three patient populations ([1] concordantly high API and CPO [best prognosis], [2] discordant API and CPO [intermediate prognosis], [3] concordantly low API and CPO [worst prognosis]) with incremental risk of the combined end‐point of death, left ventricular assist device or transplant at 6 months. (B) API and CPO measurements depicted utilizing pressure−volume loops. C. Pressure−volume loops demonstrating the relationship and utility of API and CPO in different clinical states.

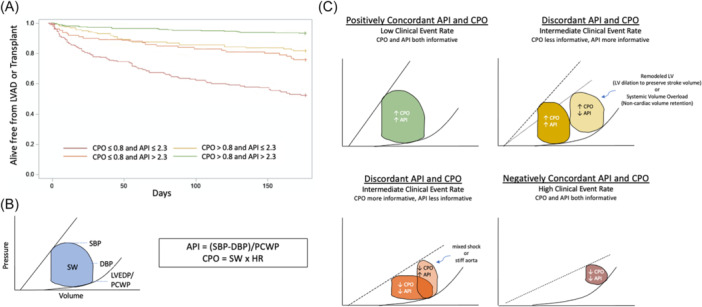

**Abbreviations**
AMI: acute myocardial infarction
API: aortic pulsatility index
AUC: area under the curve
CI: cardiac index
CO: cardiac output
CPO: cardiac power output
CSWG: Cardiogenic Shock Working Group
HF: heart failure
LVAD: left ventricular assist device
MAP: mean arterial pressure
mPAP: mean pulmonary artery pressure
OHT: orthotopic heart transplant
OR: odds ratio
PADP: pulmonary artery diastolic pressure
PAPI: pulmonary artery pulsatility index
PASP: pulmonary artery systolic pressure
PCWP: pulmonary capillary wedge pressure
RAP: right atrium pressure
RHC: right heart catheterization
ROC: receiver operator characteristic
SBP: systolic blood pressure
SW: stroke work
UNOS: United Network of Organ Sharing

## 1 INTRODUCTION

Heart failure (HF) is a serious public health concern in the United States, affecting over 6.5 million patients, with 650,000 new cases diagnosed annually.^1^ Despite progress in treatment, an estimated 1%−10% of patients progress to advanced HF with dismal 5‐year survival rates among Stage C and D patients of 75% and 20%, respectively.^2^, ^3^ Thus, accurate prognostication is of great importance to allow timely referral for consideration of advanced therapies, namely orthotopic heart transplantation (OHT) and durable mechanical circulatory support (left ventricular assist device [LVAD]).

Invasive hemodynamics are fundamental to characterization and prognostication in advanced HF. Nevertheless, it is unclear which hemodynamic parameters are most prognostic, particularly with respect to long‐term outcomes such as the need for advanced therapies. Moreover, an important consideration with the advent of advanced hemodynamic parameters such as cardiac power output (CPO), pulmonary artery pulsatility index (PAPI), and aortic pulsatility index (API) is whether these parameters in isolation or in combination can be used for improved risk stratification and long‐term prognostication.

An exploratory analysis of the ESCAPE trial, for example, showed that filling pressures, including right atrial pressure (RAP) and pulmonary capillary wedge pressure (PCWP), were significantly associated with the primary outcome of the combined risk of death, cardiovascular hospitalization and OHT, but cardiac index (CI) was not.^4^, ^5^ Since then, numerous novel indices have been reported to show prognostic value. For example, PAPI, calculated as the ratio of pulmonary artery pulse pressure to RAP, less than 1.7, was found to be a significant predictor of death or hospitalization at 6 months.^6^ Subsequently, Belkin et al. found that the API, calculated as (systolic‐diastolic blood pressure)/PCWP, greater than 1.45 in patients with stage D HF and greater than 2.9 in the less‐acute ESCAPE cohort was associated with decreased risk of death and need for LVAD or OHT at 30 days and 6 months, respectively.^7^, ^8^ CPO calculated as cardiac output (CO) × mean arterial pressure (MAP)/451, less than 0.6, has been shown to correlate strongly with in‐hospital mortality in a patient population of cardiogenic shock secondary to acute myocardial infarction (AMI) but did not show a correlation with risk of death/LVAD/OHT at 6 months in a less acutely ill patient population.^7^, ^9^

Application of various hemodynamic variables and risk scores for prognostication is challenging in practice due to substantial variability in trial design and study cohorts, as most data were derived and validated in selective clinical trial populations or retrospective observational single‐center or multicenter studies. Here, we report that advanced hemodynamic parameters provide important prognostic information in a “real‐world” contemporary US‐based cohort of HF patients across several centers spanning a large geographic range.

## 2 METHODS

The PRognostic Evaluation During Invasive CaTheterization for Heart Failure (PREDICT‐HF) is a registry comprised of retrospective patient data from 10 member institutions of the Southeast Future Leaders in Growing Heart Failure Therapies (SE‐FLIGHT) program (Supporting Information Material). In an effort to define the relative prognostic potential of both standard and advanced hemodynamic variables with the association of future HF events in a cohort of chronic and acute on chronic patients with systolic dysfunction, the PREDICT‐HF hemodynamic repository was queried. Those with discordant API and CPO had similar freedom from endpoint The study was approved by the central Institutional Review Board (IRB) at Atrium Heath (IRB #02‐21‐06E) with independent IRB approval and data sharing agreements subsequently obtained for all participating sites. Atrium Health served as the data coordinating center and performed all statistical analysis. Charts were reviewed from January 1, 2013, to December 31, 2019, and patients with chronic or acute on chronic HF symptoms undergoing isolated right heart catheterization (RHC) were included in the analysis. Patients with AMI, de novo HF, and those undergoing concomitant left heart catheterization for revascularization or valvular procedures, such as transcatheter edge‐to‐edge repair or transcatheter aortic valve replacement, at the time of RHC, were excluded. Those undergoing RHC solely for the assessment of pulmonary hypertension were also excluded. A full list of inclusion and exclusion criteria is included in the Supporting Information Material. Patient demographics, laboratory variables, vital signs, and hemodynamic variables were collected. The primary cumulative endpoint for this analysis was survival to advanced surgical HF therapy (LVAD or OHT) or death within 6 months of RHC.

### 2.1 Statistical analysis

Demographics, past medical history, baseline laboratory, and hemodynamic values were summarized as frequencies and percentages for categorical variables and as means (±standard deviation) for continuous variables and compared between patients that received medical management versus advanced therapies or death with either Student *t*‐tests or Mann−Whitney *U* (Wilcoxon) tests depending on normality as determined by Shapiro−Wilk tests for continuous variables, and *χ* ^2^ or Fisher's exact tests for categorical variables. Relationships between baseline characteristics, laboratory, and hemodynamic variables were evaluated using univariate logistic regression analysis. Covariates with a *p*‐value of <.05 on univariate regression analysis and those with previously established clinical relevance were included in a multivariable logistic regression model that assessed the independent association of these traditional sociodemographic, laboratory, and hemodynamic variables with the primary outcome. To evaluate whether the novel hemodynamic variables (API and CPO) provide added prognostic information, these models were repeated with the addition of these novel parameters. Hemodynamic variables were checked for multicollinearity using Spearman's rank correlations, and two separate adjusted models were run so there were no multicollinearity issues between the traditional and more novel hemodynamic variables. Receiver operator characteristic (ROC) curves were used to determine the appropriate cut‐off values, as well as the sensitivity, specificity, correctly classified, and area under the curve values for each of the six hemodynamic variables. Kaplan–Meier time‐to‐event analysis was conducted to describe time to the composite endpoint and then tested using log‐rank tests. To assess whether the combination of API and CPO was more discriminatory for the outcome of interest, we compared logistic regression models with CPO and API cut‐offs, and discriminant ability was assessed with concordance (c) statistics. All tests were two‐tailed and considered statistically significant with a *p* < .05. All statistical analyses were performed using SAS Enterprise Guide 7.1 (SAS Institute).

## 3 RESULTS

A total of 846 patients were included for analysis. The mean age was 58.8 years old, and participants were 43% female and 50.2% African American. Baseline demographics are presented in Table 1. Of the 846 patients included, 176 (21%) met the primary endpoint, with 76 (42%) undergoing LVAD, 28 (16%) undergoing OHT, and 75 (42%) dying. Patients of male gender or having a past medical history of ventricular tachycardia were more likely to meet the primary endpoint. Laboratory values at baseline showed sodium, blood urea nitrogen, creatinine, and alanine aminotransferase were all significantly different between the two cohorts. The majority of those who met the primary outcome either underwent LVAD implantation (*n* = 76, 42%) or died (*n* = 75, 42%) as compared to those who underwent transplant (*n* = 28, 16%).

**Table 1:** Baseline demographic characteristics, laboratory, and right heart catheterization measurements of study participants.

	Study group (*N* = 846)		Transplant or LVAD or death in 6‐months (*n* = 176 [21%])		Only medical management (*n* = 667 [79%])		*p* Value
Baseline characteristics							
Age (mean ± SD)	58.8	14.5	58.8	14.5	59.2	13.6	.7735
Female	366	43.3%	61	34.1%	305	45.7%	.0050
Race							.1843
White	331	39.1%	80	44.7%	251	37.6%	
African American	425	50.2%	84	46.9%	341	51.1%	
Other	90	10.6%	15	8.4%	75	11.2%	
Past medical history							
Nonischemic cardiomyopathy	201	23.8%	56	31.3%	145	21.7%	.1165
Coronary artery disease	329	38.9%	74	41.3%	255	38.2%	.4485
Hyperlipidemia	267	31.6%	67	37.4%	200	30.0%	.0570
Peripheral vascular disease	43	5.1%	11	6.1%	32	4.8%	.4661
Obstructive sleep apnea	172	20.3%	45	25.1%	127	19.0%	.0718
Atrial fibrillation	157	18.6%	32	17.9%	125	18.7%	.7919
Ventricular tachycardia	88	10.4%	29	16.2%	59	8.8%	.0042
Baseline laboratory values (mean ± SD)							
White blood cell (10^3^/µL)	8.0	4.0	8.4	5.0	7.9	3.6	.2099
Hemoglobin (g/dL)	12.3	2.3	12.3	2.2	12.2	2.3	.6727
Platelets (10^3^/µL)	223.3	88.3	206.6	88.8	228.6	87.5	.0056
Sodium (mmol/L)	138.9	4.1	138.1	4.7	139.2	3.9	.0114
Potassium (mmol/L)	4.2	0.6	4.2	0.6	4.2	0.6	.6684
Blood urea nitrogen (mg/dL)	30.0	20.4	35.6	25.1	28.2	18.4	<.0001
Creatinine (mg/dL)	1.7	1.5	1.9	1.8	1.6	1.3	.023
Albumin (g/dL)	3.6	0.6	3.5	0.6	3.7	0.6	.0688
Alanine aminotransferase (U/L)	57.2	209.8	105.0	373.5	40.8	102.3	.0014
Aspartate aminotransferase (U/L)	65.4	303.8	128.4	562.4	43.6	116.2	.0717
Baseline hemodynamics (mean ± SD)							
Right atrial pressure (mmHg)	11.0	6.6	13.0	7.6	10.5	6.2	<.0001
Systolic blood pressure (mmHg)	122.0	21.9	113.7	16.5	124.2	22.6	<.0001
Diastolic blood pressure (mmHg)	72.8	13.0	71.3	12.5	73.2	13.1	.074
Mean arterial pressure (mmHg)	89.2	14.0	85.4	12.2	90.2	14.3	<.0001
Mean pulmonary arterial pressure (mmHg)	31.7	11.6	36.2	10.6	30.5	11.6	<.0001
Pulmonary artery systolic pressure (mmHg)	47.1	17.1	52.6	15.4	45.6	17.3	<.0001
Pulmonary artery diastolic pressure (mmHg)	22.6	9.2	26.1	8.5	21.7	9.2	<.0001
Pulmonary capillary wedge pressure (mmHg)	20.0	9.4	24.6	9.6	18.8	9.0	<.0001
Pulmonary artery pulsatility index	3.2	2.9	3.0	2.7	3.2	2.9	<.0001
Aortic pulsatility index	3.2	2.3	2.1	1.5	3.5	2.4	<.0001
Cardiac power output	1.0	0.5	0.8	0.3	1.1	0.5	<.0001
FICK cardiac output (L/min)	5.1	2.2	4.1	1.5	5.4	2.3	<.0001
FICK cardiac index (L/min/m^2^)	2.5	1.3	2.1	1.3	2.7	1.3	<.0001

Abbreviation: LVAD, left ventricular assist device.

### 3.1 Hemodynamic variables associated with surgical HF therapies or death

**Figure 1:**
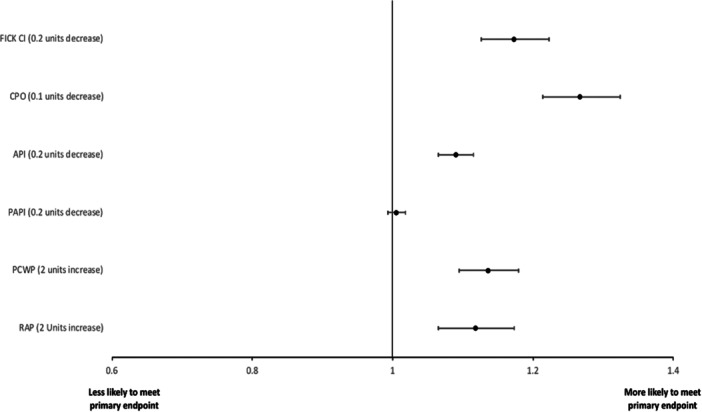
Univariate analysis of hemodynamic parameters. API, aortic pulsatility index; CI, cardiac output; CPO, cardiac power output; PAPI, pulmonary artery pulsatility index; PCWP, pulmonary capillary wedge pressure; RAP, right atrial pressure.

As seen in Table 1, most of the hemodynamic parameters were significantly different between the two groups, with the patients who were maintained on medical therapy having, on average, a lower RAP, mean pulmonary artery pressure (mPAP), pulmonary artery systolic pressure (PASP), pulmonary artery diastolic pressure (PADP), and PCWP; and higher systolic blood pressure (SBP), MAP, mPAP, PASP, PADP, PCWP, API, PAPI, CPO, Fick CO, and Fick CI were significantly different between the two groups. In a univariate analysis, RAP, PCWP, API, CPO, and Fick CI were all shown to be associated with the primary outcome (Figure 1). Results from multivariable models assessing the association between traditional and advanced hemodynamic variables with outcome of interest are presented in Table 2. In the model incorporating traditional variables, PCWP (OR: 1.10, 95% CI: 1.04−1.15, *p* < .001) and CI (OR: 0.86, 95% CI: 0.81−0.92, *p* < .001) were significantly associated with the primary outcome. When the advanced hemodynamic variables were added to the model, all three variables were shown to be significant, with CPO (OR: 0.76, 95% CI: 0.71−0.83, *p* < .001) and API (OR: 0.94, 95% CI: 0.91−0.96, *p* < .001) having a more robust association as compared to PAPI (OR: 1.02, 95% CI: 1.00−1.03, *p* .027).

**Table 2:** Multivariate analyses of hemodynamic variables.

	OR	95% CI		*p* Value
Multivariable analysis Model‐I				
AoPI (0.2 units decrease)	1.07	1.04	1.10	<.001
PAPI (0.2 units decrease)	0.98	0.97	1.00	.0267
CPO (0.1 units decrease)	1.31	1.21	1.42	<.001
Multivariable analysis Model‐II				
PCWP (2 units increase)	1.10	1.04	1.15	<.001
RAP (2 units increase)	0.99	0.93	1.07	.8551
FICK CI (0.2 units decrease)	1.16	1.09	1.23	<.001

Abbreviations: CPO, cardiac power output; PAPI, pulmonary artery pulsatility index; PCWP, pulmonary capillary wedge pressure; RAP, right atrial pressure.

### 3.2 Cut‐off derivation and predictive value of hemodynamic variables

**Figure 2:**
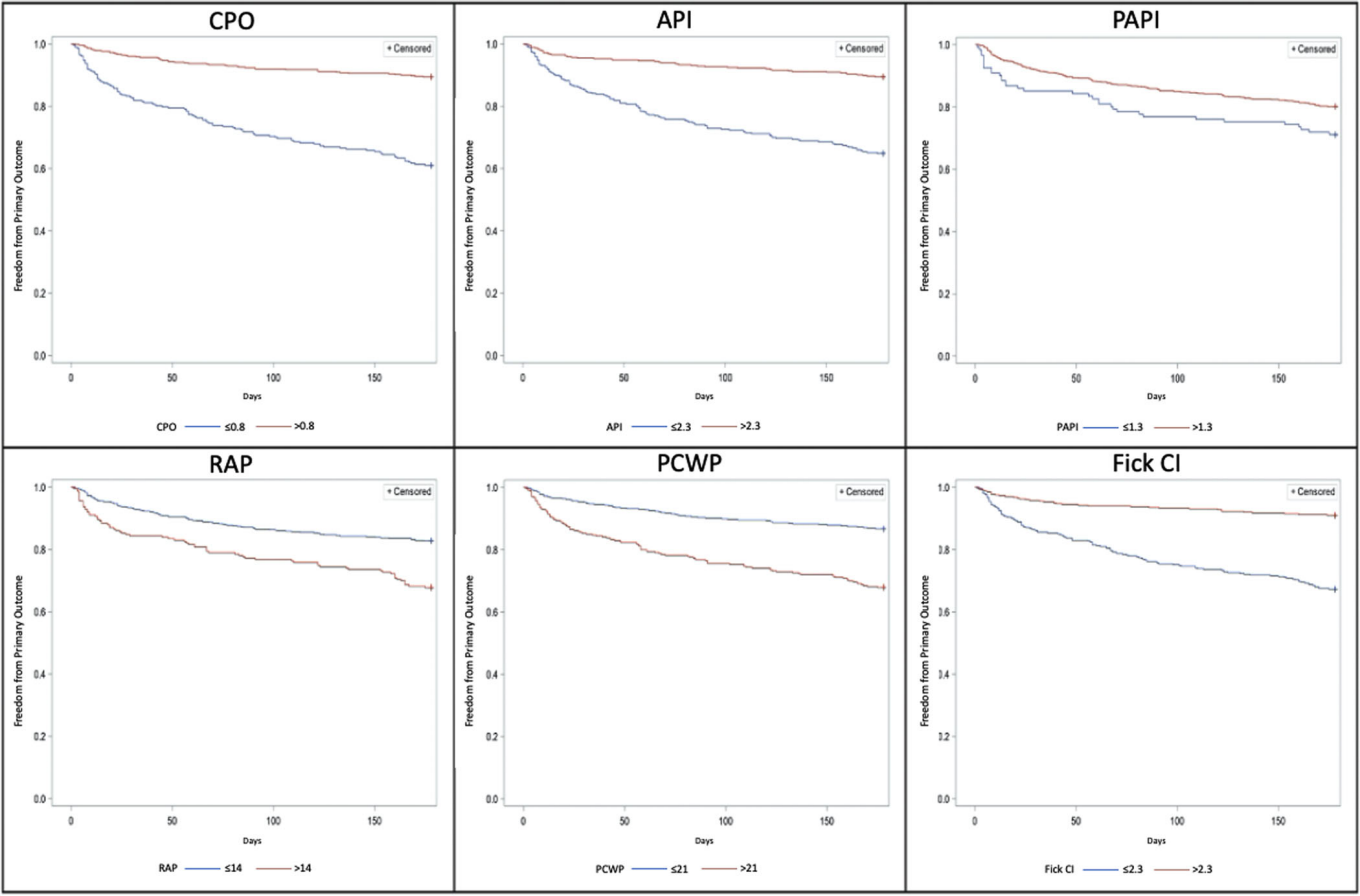
Predictive value of hemodynamic variables—Kaplan−Meier curves. API, aortic pulsatility index; CI, cardiac output; CPO, cardiac power output; PAPI, pulmonary artery pulsatility index; PCWP, pulmonary capillary wedge pressure; RAP, right atrial pressure.

We subsequently used ROC analysis to identify cut‐off values for each of the hemodynamic variables that were shown to be significant in our multivariable model (Supporting Information analysis). Kaplan−Meier survival analysis showed that CPO (cut‐off 0.8, *p* < .001), API (cut‐off 2.3, *p* < .001), PAPI (cut‐off 1.3, *p* < .016), RAP (cut‐off 14 mmHg, *p* < .001), PCWP (cut‐off 21 mmHg, *p* < .001), and Fick CI (cut‐off 2.3, *p* < .001) were all predictive of the primary outcome (Figure 2).

When API and CPO were used in combination, patients with positively concordant API and CPO had the best freedom from endpoint (94.7%), whilst those with negatively concordant API and CPO had the worst freedom from endpoint (61.5%, *p* < .001). Those with discordant API and CPO were in between (high API and low CPO 83.7%; low API and high CPO 89.7%) and had similar freedom from endpoint to each other and represented an intermediate prognosis cohort. A model with API and CPO together performed better in predicting advanced treatments or death within 6 months with a *c*‐statistic of 0.75 (95% CI: 0.71−0.79) than a model with either API (*c*‐statistic 0.68, 95% CI: 0.64−0.72) or CPO (*c*‐statistic 0.70, 95% CI: 0.66−0.74) alone.

## 4 DISCUSSION

In this contemporary, multi‐institutional analysis of all‐comers with HF who underwent RHC, advanced hemodynamic parameters were found to be highly prognostic at predicting the 6‐month need for advanced therapies or death. The predominant findings from our analysis are as follows: (1) API and CPO are highly prognostic of the need for advanced surgical therapies or death at 6 months, and (2) There is additive value for risk stratification using API and CPO concomitantly when compared to API or CPO alone.

Identification of the prognostic potential of both traditional and advanced hemodynamic variables has been historically challenging.^10^, ^11^, ^12^ CI, once thought to be the quintessential hemodynamic parameter, has shown mixed results for prognostication.^10^, ^12^, ^13^ Even in the more contemporaneous era, CI continues to have mixed results. In a subanalysis of the ESCAPE trial, residual congestion and not CI were predictive of 6‐month events.^5^ In a robust registry from the Veteran Affairs Administration, Thermodilution CI was more predictive of mortality than estimated Fick CI, with poor agreement between the two measurements.^14^ Conversely, the congestive profile of the patient has shown to be a more reliable prognostic marker.^10^, ^12^, ^13^, ^15^ Both an elevated RAP and PCWP portend a poor prognosis and need for advanced therapies or death.^5^ CPO similarly has shown mixed results in terms of prognostication. In the Shock trial, CPO was found to be the strongest predictor of mortality. However, more recently, CPO failed to discriminate clinical events in the Cardiogenic Shock Working Group analysis (CSWG).^9^, ^16^, ^17^

The heterogeneity of the hemodynamic parameters is largely driven by heterogeneous patient populations, patient acuity and comorbidities, and different eras of clinical medicine, including background device and medication optimization. When patients are in cardiogenic shock, it is often more straightforward to identify patient acuity. However, this task is often challenging in ambulatory patients with chronic HF. Hemodynamic parameters may have different relative weights depending on the stage and severity of the disease as well as the mechanism of shock. For example, the Shock trial, which showed CPO to be highly prognostic, was conducted in a patient population of acute MI shock, whereas the CSWG analysis was conducted in a patient population of cardiogenic shock related to both AMI and chronic HF.^9^, ^17^

We postulate that the chronicity of shock and the patient's physiologic response may influence the discriminatory potential of some of the hemodynamic parameters. In response to an acute drop in contractility, such as following AMI, the end‐systolic pressure−volume relationship shifts downward (reduction in end‐systolic elastance Ees), leading to an immediate reduction in stroke volume, stroke work (SW), CO, and CPO. If remodeling occurs, activation of the renin−angiotensin−aldosterone axis leads to the retention of salt and water, leading to a rightward shift along the end‐diastolic pressure−volume relationship.^18^ Depending on the afterload and the contractile state of the patient, this often normalizes stroke volume, SW, CO, and CPO. Under these conditions, CPO may not fully reflect the clinical state of the patient, whereas API would still be prognostic.^19^ Conversely, API can be influenced by the aortic properties and vascular resistance, which can influence the pulse pressure independently from CO. Thus, in settings of mixed shock or in patients with stiff aortic vasculature, API may be high and not fully reflect the clinical state whereas CPO may be more prognostic in this context. Patients with a discordant API and CPO, regardless of which value is low (high CPO with low API or high API with low CPO), portends an intermediate prognosis compared to patients with concordantly low API and CPO. The simultaneous use of API and CPO may thereby overcome the individual limitations of each parameter in isolation.^20^ A negatively concordant low API and CPO reflects a rightward and downward shifted pressure−volume loop and reflects a patient who is both congested and in a low output state.

The 2022 AHA/ACC/HFSA Heart Failure guidelines have downplayed the role of continuous pulmonary artery pressure monitoring in routine patient care, bestowing a class II recommendation.^21^ The guideline recommendations largely reflect the disparate literature surrounding the role of hemodynamic monitoring in the day‐to‐day management of a hospitalized HF patient across the spectrum of continuity. A complete hemodynamic assessment still has an important role in the prognostication and suitability of advanced therapies. Herein, we show that API and CPO, both in isolation and in combination, have a key role in assessing the need for advanced therapies. Since October 2018, the current United Network of Organ Sharing (UNOS) allocation system heavily relies on CI, SBP, and PCWP to predict waitlist mortality. Additional studies are needed to determine if the addition of advanced hemodynamic parameters can improve risk assessment at the time or transplant as it relates to status designation at the time of listing.^22^

## 5 LIMITATIONS

There are several limitations in this analysis. This is a retrospective cohort study, and as such there are inherent limitations and residual confounding that cannot be excluded with this type of analysis. Predictive analysis is best performed with a prospective data collection, given the potential for unaccounted for bias. There was a non‐standard distribution of patients in the cohort from the different participating centers, both individually and geographically. When reviewing center‐level data, however, there was a lower clinical event rate in the largest contributing center, and as such, any bias would be towards the null hypothesis, which, in turn, strengthens the validity of the results. The inclusion of several centers with differing practice patterns may have effects on patient management, especially regarding the delivery of advanced therapeutic options. The variability in practice patterns is even further exacerbated by the inclusion period straddling the changes to the UNOS allocation system in 2018. We sought to account for this by our use of the composite endpoint that incorporated both OHT and LVAD as well as mortality. From a statistical approach, hemodynamic variables were checked for multicollinearity using Spearman's rank correlations, and two separate adjusted multivariable models were needed to avoid multicollinearity issues between the traditional and advanced hemodynamic variables.

Lastly, we chose to test the thresholds of the parameters in the same data set from which they were derived to establish the appropriate cut points for a “real world” patient population of variable patient acuity from multiple different centers. Given the variability in patient acuity together with our modest sample size, a validation and derivation cohort with this data set was felt to be insufficient to test our hypothesis as it would potentially underestimate the prognostic potential of these parameters. Future work should include a prospective data collection with a separate derivation and validation set.

## 6 CONCLUSIONS

The advanced hemodynamic parameters of API and CPO were highly correlated with the need for advanced surgical therapies or death, and when used in combination, they had added predictive value. Incorporation of these novel hemodynamic parameters in routine risk stratification may assist with more timely and appropriate referrals for advanced therapies. Further prospective studies are needed to validate these hemodynamic parameters and elucidate their role in patients with advanced HF.

## AUTHOR CONTRIBUTIONS

All listed authors contributed to the concept and design of the study and reviewed the analysis and interpretation of the data in addition to the writing of the manuscript with final approval of the manuscript.

## CONFLICT OF INTEREST STATEMENT

J. G. is a speaker for Abbott, Abiomed, Medtronic, and CH Biomedical. The remaining authors declare no conflict of interest.

## Supporting information

Supporting information.

## Data Availability

The data that support the findings of this study are available from the corresponding author upon reasonable request.
