# Supplementary material for: PREDICT HF: Risk stratification in advanced heart failure using novel hemodynamic parameters
Source: Clin Cardiol. 2024 Jun 5;47(6):e24277. doi: 10.1002/clc.24277 (PMC11151004; doi:10.1002/clc.24277)
Supplement: Supplementary file 1 — Supporting information. [file CLC-47-e24277-s001.docx]

**Supplemental Material**

**Participating Member Institutions of SE-FLIGHT Group**

Atrium Health Carolinas Medical Center

Atrium Health Cabarrus

Cleveland Clinic Florida

Duke University Medical Center

Inova Heart and Vascular Institute

May Clinic in Florida

Medstar Washington Hospital Center

University of Chicago

University of Florida

University of South Florida

**Study Criteria**

**Inclusion Criteria:**

Adults ≥18 years of age

NYHA class 2 through 4 symptoms

Diagnosis of heart failure

Right heart catheterization performed at one of the member institutions of SE-FLIGHT Group

**Exclusion Criteria:**

- 1. Chronic inotropes
  2. Mechanical Circulatory Support
  3. Heart transplant
  4. Acute Type I myocardial infarction or revascularization
  5. Concomitant Valvular intervention at the time of RHC
  6. Right heart catheterization solely for the purpose of pulmonary hypertension assessment
  7. End Stage Renal Disease on hemodialysis
  8. Uncorrected complex congenital heart disease
  9. Acute pulmonary embolism
  10. Cardiac amyloidosis
  11. Other disease with life expectancy < 1 year
  12. Noncardiogenic shock (including tamponade)
  13. Mitral stenosis (> moderate)
